# Supplementary material for: Enhancing the Catalytic Activity of Type II L-Asparaginase from Bacillus licheniformis through Semi-Rational Design
Source: Int J Mol Sci. 2022 Aug 26;23(17):9663. doi: 10.3390/ijms23179663 (PMC9456134; doi:10.3390/ijms23179663)
Supplement: Supplementary file 1 [file ijms-23-09663-s001.zip › ijms-1884343-supplementary.pdf]

Supplementary Material

Table S1. Primers used in this study.

Residue Sites	Name	Primer (5′-3′)
L203	L203-F	GGTGGTGMNNAATGACCGGATTGCTGCGGCAA
	L203-R	GGTCATTNNKCACCACCATGGTTCCTTTCCCG
K218	K218-F	ACCCATMNNACAGCTCCTGACACTTTCAAGTCG
	K218-R	GGAGCTGTNNKATGGGTTTTCGTCACATATCTTGC
T223	T223-F	CAGCTCCTGACMNNTTCAAGTCGGAAGAAATGGGG
	T223-R	GAANNKGTCAGGAGCTGTCGTATGGGTTTTCG
T325	T325-F	TCTACACGCMNNGGCAGCGGGGTTGTCACGCC
	T325-R	CTGCCNNKGCGTGTAGAACGCACGACTGCCGC
A180	A180-F	CGGGCCTMNNAACTTGTACAATGCTGTAAAGGTCG
	A180-R	ACAAGTTNNKAGGCCCGTCGGCTCCAATCGCT
E102/R123	E102-F	CAGTGGAMNNCAAGTGGCAAACATCGGAAGTC
	E102-R	CCACTTGNNKTCCACTGATATTCGCGATATCCT
	R123-F	GAGCAAGMNNACTGCGAAGCTTTTAGCTTCAGA
	R123-R	TCGCAGTNNKCTTGCTCAATTTCAGGAGCGTT
V104/R57	V104-F	GTGGAGAACAAMNNGCAAACATCGGAAGTCAGAATATG
	V104-R	TGCNNKTTGTTCTCCACTGATATTCGCGATAT
	R57-F	GCCCAACATAMNNATTTTGGCTACAGGGGGGAC
	R57-R	AAATNNKTATGTTGGGCAGACGGTCATTTCCT

Notes: The underlines represented the mutation sites; N indicated any of the following A, T, G, or C; K indicated G or T; M indicated A or C.

Table S2. Enzymatic activity and specific activity of the wild-type and positive mutants.

Enzymes	Enzymatic Activity（IU/ml）	Specific activity (IU/mg)
WT	14.78±1.43	198.40±2.92
E102I/V104L	41.24±2.36	486.83±9.52
E102I/A180R	40.40±3.45	426.15±12.64
E102I/T223A	41.43±1.77	454.21±19.32
E102I/T325C	39.46±2.59	517.76±13.92
V104L/A180R	39.37±2.88	432.57±16.88
V104L/T223A	33.26±3.96	418.35±7.44
V104L/T325C	45.37±3.36	430.99±8.64
A180R/T223A	47.44±2.56	479.11±17.68
A180R/T325C	41.68±3.59	313.98±7.28
T223A/T325C	36.54±2.97	355.51±13.76
E102I/T223A/T325C	51.73±6.39	705.45±15.21
E102I/T223A/A180R	35.57±1.84	480.61±5.84
A180R/T223A/E102I	36.69±5.76	522.93±13.75
A180R/T223A/T325C	39.57±2.71	646.54±9.91
E102I/A180R/T223A/T325C	53.74±5.71	760.64±19.40
E102I/V104L/T223A/T325C	55.29±4.44	812.32±19.07
V104L/A180R/T223A/T325C	55.47±7.23	794.75±11.5
E102I/A180R/T223A/T325C	53.74±5.71	790.61±17.95
